# Bioremediation of petroleum hydrocarbons from crude oil-contaminated soil with the earthworm: *Hyperiodrilus africanus*

**DOI:** 10.1007/s13205-015-0298-1

**Published:** 2015-04-09

**Authors:** O. A. Ekperusi, F. I. Aigbodion

**Affiliations:** Environmental Quality Management Programme, Department of Animal and Environmental Biology, Faculty of Life Sciences, University of Benin, Benin City, Nigeria

**Keywords:** Vermiremediation, Crude oil, Earthworms, *Hyperiodrilusafricanus*, Nigeria

## Abstract

A study on the bioremediation potentials of the earthworm *Hyperiodrilusafricanus* (Beddard) in soil contaminated with crude oil was investigated. Dried and sieved soils were contaminated with 5 ml each of crude oil with replicates and inoculated with earthworms and monitored daily for 12 weeks. Physicochemical parameters such as pH, total organic carbon, sulfate, nitrate, phosphate, sodium, potassium, calcium and magnesium were determined using standard procedures. Total petroleum hydrocarbon (TPH) was determined using atomic absorption spectrophotometer (AAS), while BTEX constituents and earthworms tissues were analyzed using Gas Chromatography with Flame Ionization Detector (GC-FID). The results showed that the earthworm significantly enhanced the physicochemical parameters of the contaminated soil resulting in a decrease of the total organic carbon (56.64 %), sulfate (57.66 %), nitrate (57.69 %), phosphate (57.73 %), sodium (57.69 %), potassium (57.68 %), calcium (57.69 %) and magnesium (57.68 %) except pH (3.90 %) that slightly increased. There was a significant decrease in the TPH (84.99 %), benzene (91.65 %), toluene (100.00 %), ethylbenzene (100.00 %) and xylene (100.00 %). Analyses of the tissues of the earthworm at the end of the experiment showed that the earthworms bioaccumulated/biodegraded 57.35/27.64 % TPH, 38.91/52.73 % benzene, 27.76/72.24 % toluene, 42.16/57.85 % ethylbenzene and 09.62/90.38 % xylene. The results showed that the earthworms *H. africanus* could be used to bioremediate moderately polluted soil with crude oil contamination in the Niger Delta region of Nigeria.

## Introduction

The petroleum industry in Nigeria is the largest in the West African region and the second largest in Africa after Algeria. Nigeria has a total of 159 oil fields and 1481 wells in operation (NDES 1997). According to the Nigerian National Petroleum Corporation (NNPC) 2013 Annual Statistical Bulletin on the oil and gas industry, a total crude oil and condensate production for the year was 852,776,653 barrels, giving a daily average of 2.27 million barrels per day (mb/day). These figures put Nigeria in the fifteenth position in relation to the global oil producing nation and the sixth largest exporter of crude oil worldwide (NNPC 2013).

All activities surrounding the oil sector such as exploration, drilling, transportation, refining and consumption of oil and its associated products result in the spilling of oil and refine products into the environment. In 2013 alone, the NNPC reported a total of 2256 line breaks on NNPC pipelines resulting in a loss of 181.67 million tonnes (mt) of petroleum products worth about N21.48 billion, with 34 cases of fire incidents during the year under review (NNPC 2013).

Crude oil contains a wide range of compounds which are largely toxic to humans and the environment. Some authors have suggested that soil remediation standards should be based on the BTEX components in crude oil and oil products (fuels)-impacted soils (Salanitro et al. 1997). Although BTEX are known to vapourize in contaminated sites, they can remain locked in soil for months and even years as reported by the United Nations Environment Program assessment in Ogoniland (UNEP 2011), hence they need special attention in crude oil-contaminated soil. Each of these compounds or their combination poses a serious concern to human health, living organisms and the environment. Benzene is a notorious cause of bone marrow failure. Substantial quantities of epidemiologic, clinical, and laboratory data link benzene to aplastic anemia, acute leukemia, and bone marrow abnormalities (Kasper et al. 2004) and myelodysplastic syndrome (Smith 2010). Human exposure to benzene is a global health problem. Benzene targets liver, kidney, lung, heart and the brain and can cause DNA strand breaks, chromosomal damage, etc. Benzene causes cancer in animals including humans and has been shown to cause cancer in both sexes of multiple species of laboratory animals exposed via various routes (Huff 2007; Rana and Verma 2005). The United States Environmental Protection Agency (EPA) has classified benzene as a known human carcinogen for all routes of exposure (EPA 2012). The central nervous system (CNS) is the primary target organ for toluene toxicity in both humans and animals for acute and chronic exposures (EPA 2012). Animal studies have reported effects on the blood, liver, and kidneys from chronic inhalation exposure to ethylbenzene. Exposure to ethylbenzene by inhalation resulted in an increased incidence of kidney and testicular tumors in rats, and lung and liver tumors in mice (EPA 2000). Ethylbenzene is classified as a possible carcinogen by the International Agency for Research on Cancer (IARC 2012).

Beside, the use of chemicals and mechanical methods for cleaning oil in the environment, one of the most promising bioremediation technologies is the use of earthworms in a processed specifically known as vermiremediation.

The idea to use earthworms in vermicomposting of gardens and crop enhancement has been known for centuries, but its application in bioremediation according to available records was incidentally discovered after the Seveso chemical plant explosion in 1976 in Italy, when a vast area was contaminated with extremely toxic chemical such as 2, 3, 7, 8-tetrachlorodibenzo-p-dioxin (TCDD). Several fauna perished except for some species of the earthworms that survived. Earthworms which ingested TCDD-contaminated soils were shown to bioaccumulate dioxin in their tissues and concentrated it on an average of 14.5-fold (Satchell 1983).

Several researches have established the potentials of earthworms to bioremediate crude oil and other petrochemicals from laboratory and field trials polluted soil. Ma et al. (1995) studied the influence of earthworm species *Lumbricus rubellus* on the disappearance of spiked PAHs, phenanthrene and fluoranthene (100 μg/kg of soil), and found that the losses of both PAHs occurred at a faster rate in soils with earthworms than the soil without worms. After 56 days (8 weeks), 86 % of the phenanthrene was removed.

Other studies have also shown that oil-polluted soils can be efficiently bioremediated, leading to a reduction of toxic potency (Van Gestel et al. 2001). Martin-Gil et al. (2007) also studied the use of earthworm *Eisenia fetida* and vermicomposting in the treatment of high-molecular weight hydrocarbons asphaltenes from the prestige oil spill. Earthworms mineralized the asphaltenes, thus eliminating it from the system. Sinha et al. (2008) studied the remedial action of earthworms on PAHs contaminated soils obtained from a former gasworks site in Brisbane, Australia. Results showed that the earthworms could remove nearly 80 % of the PAHs as compared to just 47 and 21 % where it was not used and only microbial degradation occurred. Ameh et al. (2013) investigated the use of earthworms (*Eudriluseugeniae*) for vermi-assisted bioremediation of petroleum hydrocarbon-contaminated mechanic workshop soils. After 35 days of treatment, earthworm inoculation affected a higher drop in total petroleum hydrocarbon contents as compared to the samples without worms, indicating that earthworms may be used as biocatalysts in the bioremediation process.

*Hyperiodrilusafricanus* is a species of earthworms widely distributed in humid tropical Africa (Lavelle et al. 1999). Populations of this species are found throughout West (Ivory Coast, Nigeria) and Central Africa (Congo, Democratic Republic of Congo, Angola) both in natural and disturbed areas derived from humid savannas and forests (Madge 1969; Omodeo 1954; Tondoh and Lavelle 2005).

Although other species of earthworms have been used for bioremediation, literature on the bioremediation of crude oil with earthworms *H. africanus* is not available, hence this study seeks to determine the effectiveness of hydrocarbon degrading potentials of the earthworm *H.africanus* on crude oil-contaminated soil and to determine the fate of the contaminant taken by the earthworms, if they are bioaccumulated or biodegraded in the tissues of the earthworms.

## Materials and methods

### Test substrate

Top soil not exceeding a depth of five inches after clearing the vegetation cover was dug with a shovel and collected into a bucket besides the Botanical Garden, Faculty of Life Sciences, University of Benin. The collected soil was sun-dried by spreading it on a flat, clean board surface for 48 h. The dried soil was sieved using a 5 mm mesh plastic filter according to ISO standard 11268-1 (ISO 1993) to remove debris and large stones.

### Test organisms

The earthworms used for the experiment were collected around the main campus of the University of Benin immediately after rainfall while crawling around to seek shelter. All earthworms were held in a holding facility for days prior to the experiment for acclimatization purposes and were regularly checked on a daily basis to ascertain their health condition. Prior to the experiment, earthworms were identified by Stephen Owa, using methods described by Owa (1992). The crude oil used for the experiment was obtained from Chevron Escravos Terminal, Delta State, Nigeria.

### Experimental design

Four rectangular containers with cover lid and clips on both sides of the edges measuring 20 × 9 × 12 cm were purchased from the market. The containers were weighed with a Digital Sensitive Weighing balance (Scoute SE-410X0.019, Ohaus Computer, USA) and were properly labeled using tape and a permanent marker. 1 kg (ISO 1993) of the sun-dried soil was then weighed into each of the four containers using Camry Emperors scale manufactured by Dial Spring Scale, China. With the aid of a 10 ml glass beaker, 5 ml of crude oil was thoroughly mixed, into each of the four containers having 1 kg each of soil and were moistened with distilled water to the water holding capacity of the soil. The treatments with crude oil-contaminated soil were left to stay for 7 days in the laboratory exposed to the elements.

After 7 days, cow dung was freshly collected within the campus and about 50 g each of the cow dung weighed was thoroughly mixed into the containers with crude oil-contaminated soil.

Immediately after the addition of additives, earthworms were sorted out of the holding containers, washed with clean water and ten earthworms of the species *H. africanus* measured and weighed were inoculated into the crude oil-contaminated soil and the replicates except the control. A netting material cut into sizes was placed on top of each of the containers and the cover lid frame was placed on top of it to hold it firmly with the help of the clips on both sides of the containers. This is done to avoid escape of the earthworms and allow free flow of oxygen into the treatments. The setup was placed inside the laboratory and checked morning and evening on a daily basis.

### Laboratory analyses

Prior to the contamination of the soil and after contamination, samples were collected with a spatula, placed in an aluminum foil, labeled and taken to a laboratory to determine the physicochemical parameters of the soil, total petroleum hydrocarbon (TPH) and the benzene, toluene, ethylbenzene and xylene (BTEX) constituents.

**Fig. 1:**
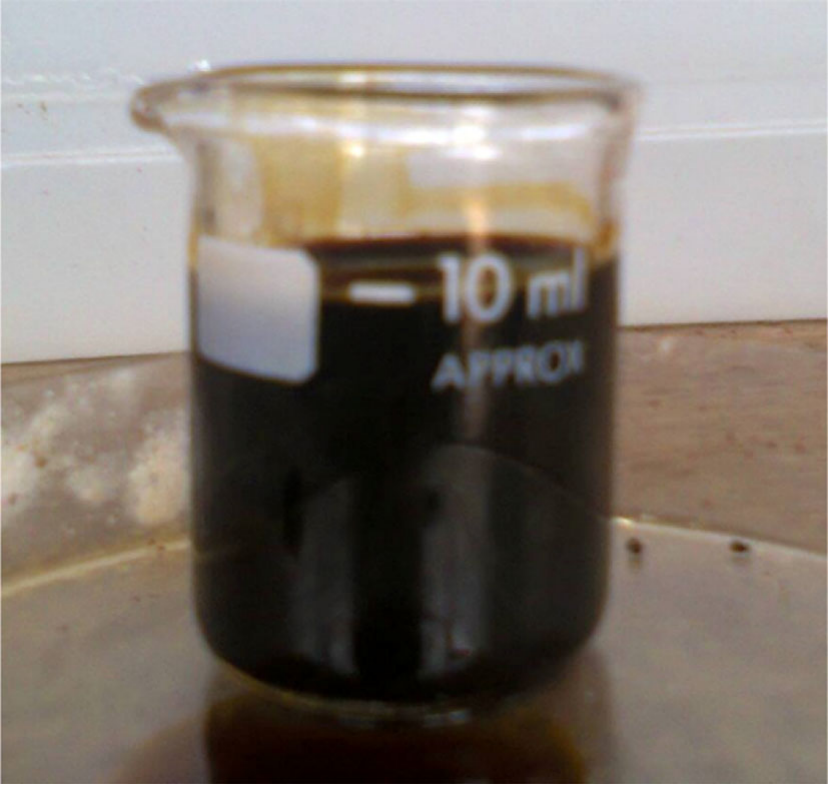
Crude oil

**Fig. 2:**
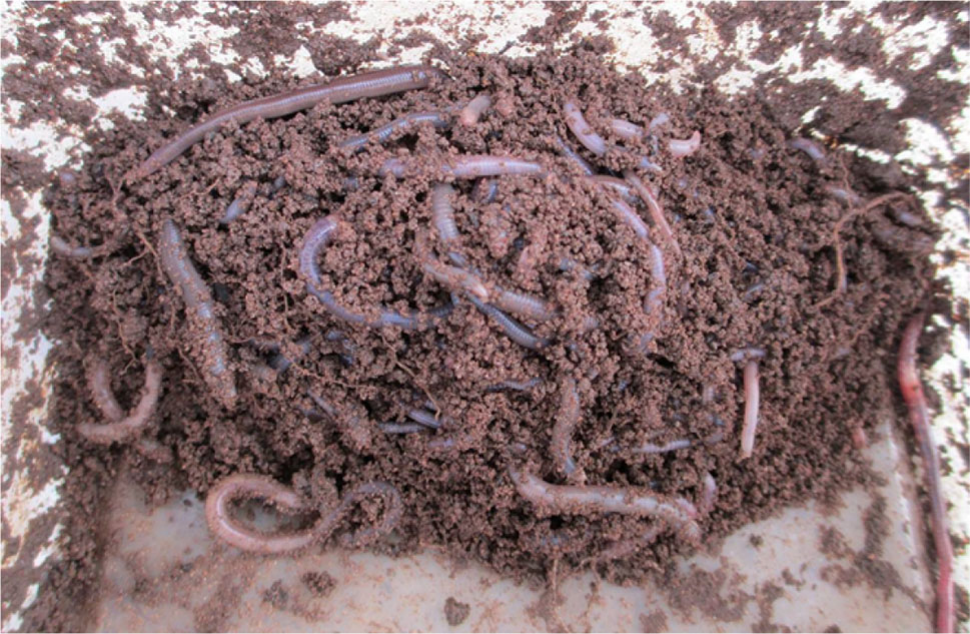
Earthworm holding container

**Fig. 3:**
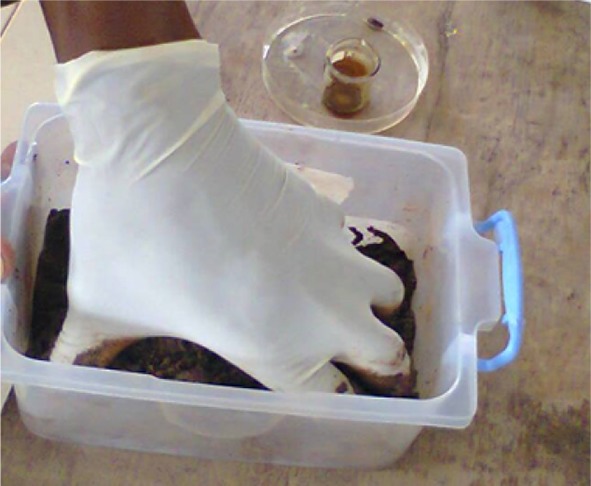
Mixing crude oil into 1 kg of soil

**Fig. 4:**
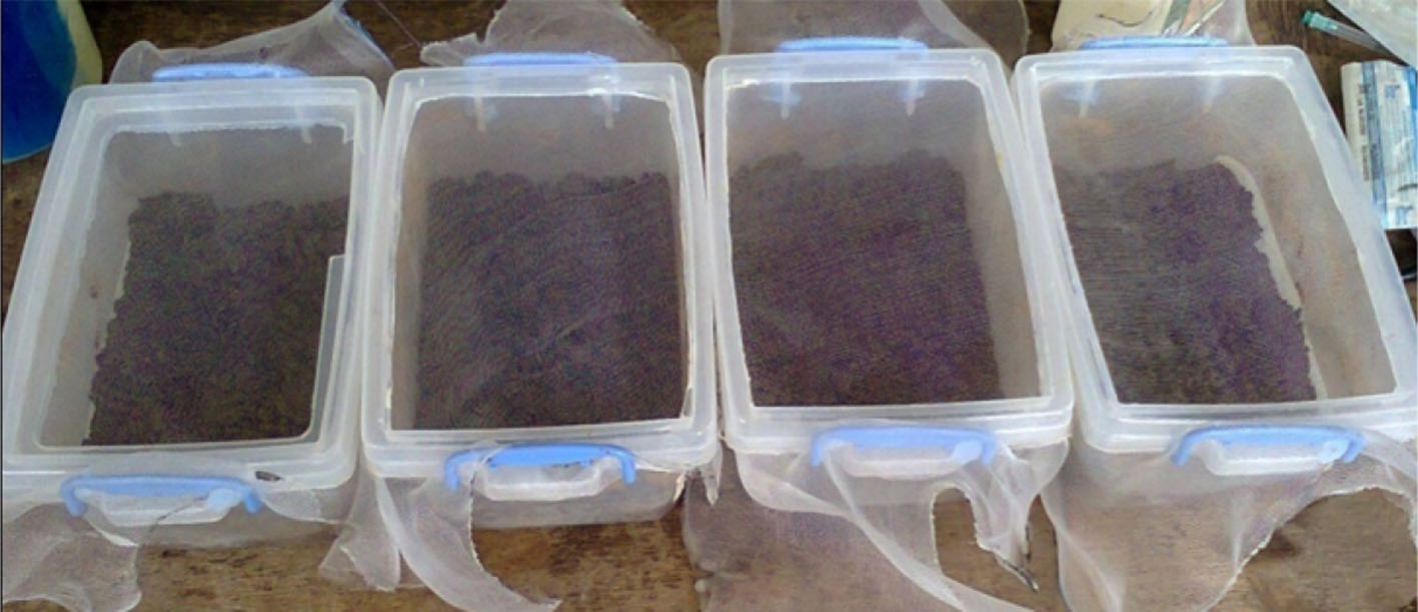
Experimental setup—three replicates and control

For every 30 days, samples of the contaminated soil from each of the treatments were collected for laboratory analyses. For each of the contaminated soils, the physicochemical parameters such as pH, total organic carbon, sulfate, nitrate and phosphate, sodium, potassium, calcium, magnesium were determined using procedures by the AOAC (2005). The TPH was determined using Atomic Absorption Spectrophotometer (AAS) as described by Miroslav and Vladimir (1999) while BTEX constituents in the crude oil (Figs. 1, 2, 3, 4) contaminated soil were determined using Gas Chromatography with Flame Ionization Detector (GC-FID) from Agilent Technologies Inc., United States. At the termination of the experiment, earthworms were analyzed to determine the TPH and BTEX in the tissues using GC-FID.

## Results

### Death and survival of earthworms

At the termination of the experiment, no death was recorded in the population of the earthworm species used for the study.

### Physicochemical parameters

**Fig. 5:**
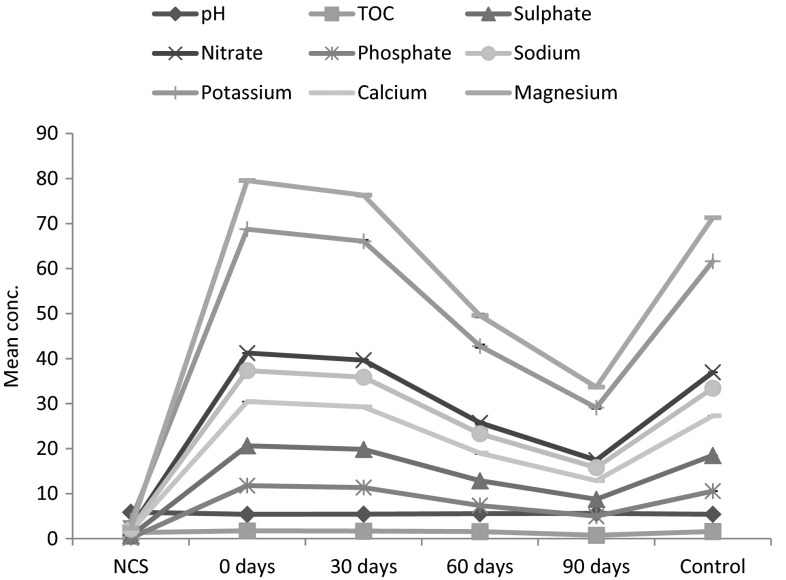
Physicochemical parameters of crude oil-contaminated soil with earthworms

The physicochemical parameters showed a variation within 90 days of the experiment. There was a gradual increased in the pH of the contaminated soil with inoculation of earthworm after 30, 60 and 90 days, (0.93, 2.78 and 3.90 %) respectively compared to the control where there is a decrease. There was a significant (*P* < 0.05) decrease in the crude oil-contaminated soil after inoculation of *H. africanus* within 30, 60 and 90 days in the total organic carbon (3.47, 9.25, 56.64 %), sulfate (3.88, 37.63, 57.66 %), nitrate (3.86, 37.63, 57.69 %), phosphate (3.74, 37.61, 57.73 %), sodium (3.89, 37.57, 57.69 %), potassium (3.91, 37.78, 57.68 %), calcium (3.91, 37.68, 57.69 %) and magnesium (4.06, 37.68, 57.68 %) compared to the control without earthworms with a 10.55 % decrease after 90 days of the study (Fig. 5).

### Total petroleum hydrocarbon bioremediation

**Fig. 6:**
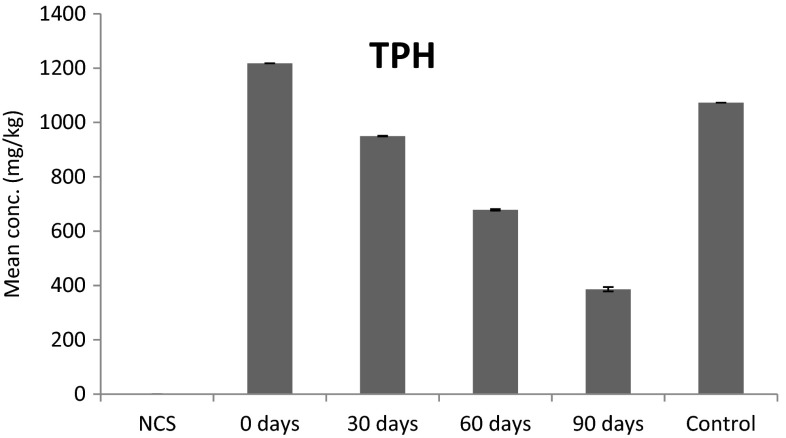
TPH in crude oil-contaminated soil with *H. africanus*

Total petroleum hydrocarbon content in crude oil-contaminated soil with *H. africanus* decreased significantly (*P* < 0.05, *F* = 16,503.64) after 30, 60 and 90 days of the experiment by 22.01, 44.29 and 68.29 %, respectively, but in the control, it only decreased by 11.87 % (Fig. 6).

### Benzene, toluene, ethylbenzene and xylene bioremediation

**Fig. 7:**
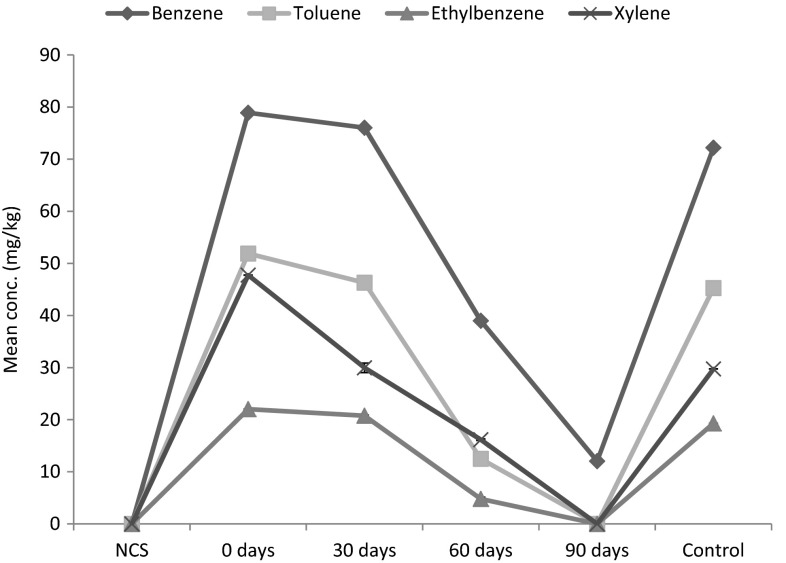
Bioremediation of BTEX in contaminated soil with earthworms

Benzene, toluene, ethylbenzene and xylene decreased with inoculation of *H. africanus* into crude oil-contaminated soil (Fig. 7). Benzene decreased by 3.65, 50.60 and 84.73 % (*F* = 181,785.77, *P* < 0.05), toluene by 10.79, 75.95 and 100.00 % (*F* = 3242.62, *P* < 0.05), ethylbenzene by 5.72, 78.24 and 100.00 % (*F* = 5217.28, *P* < 0.05) and xylene by 37.26, 66.16 and 100.00 % (*F* = 2484.56, *P* < 0.05) after 30, 60 and 90 days of the study.

### Bioaccumulation/biodegradation of TPH and BTEX

Analyses of the tissues of the earthworms, *H. africanus* at the end of the study showed that the earthworms bioaccumulated 58.05 % TPH, 48.42 % benzene, 31.86 % toluene, 63.20 % ethylbenzene and 18.14 % xylene in crude oil-contaminated soil.

To calculate the percentage biodegraded, the formula below was adopted:

$${\text{Biod}}_{\text{C}} = I_{\text{C}} {-}F_{\text{C}} {-}T_{\text{C}}$$

where Biod_C_ is the concentration of pollutant biodegraded at the end of the experiment, *I*_C_ is the initial concentration of pollutant at the beginning of the experiment, *F*_C_ is the final concentration of pollutant at the end of the experiment, and *T*_C_ is the concentration in the tissues of the earthworms at the end of the experiment

**Fig. 8:**
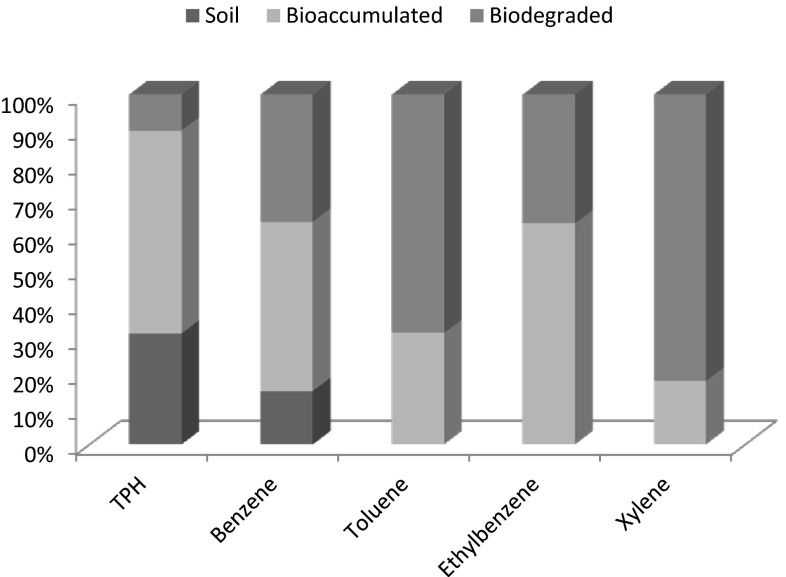
Bioaccumulation/biodegradation of TPH and BTEX in earthworms

The results revealed that the earthworms, *H. africanus*, biodegraded 10.24 % TPH, 36.34 % benzene, 68.14 % toluene, 36.80 % ethylbenzene and 81.86 % xylene (Fig. 8).

## Discussion

Bioremediation has become a universally acceptable technology for the removal of a wide range of contaminants, especially generated from the petrochemical industries from the environment.

In this present study, all the earthworms survived the concentration (5 ml) of crude oil contaminated into the 1 kg of soil and significantly degraded the TPH and BTEX hydrocarbons in the media.

Ameh et al. (2011) reported 100 % survival of earthworms in 150 g of used engine oil. At the end of our study, earthworms re-introduced into non-polluted soil continue their normal activities. The idea that some workers have used more than 5 ml of oil in other regions of the world where heavy and sour crude oil is available led to the first attempt to use 10 ml for the experiment. This was the thinking based on the fact that the Nigerian crude oil is light and maybe less toxic, but such concentration resulted in the death of the earthworms in less than 48 h. This agrees with the findings of Dorn and Salanitro (2000) which reported that light oil was the most toxic oil in their study. Heavy oils were least toxic showing LC50s of 100 % or greater after 2 ± 3 months time in either soil. The light oil in the soils was significantly more toxic than medium and heavy oils (Dorn and Salanitro 2000).

Earthworms commonly found in agricultural fields thrive at neutral pH, but can tolerate a pH from 5.0 to 8.0, (Duiker and Stehouwer 2008). The pH of the soil decreased from 5.85 to 5.39 after contamination and gradually increased from 5.39 to 5.60 after inoculation of earthworms. It is a favoured opinion that the activities of earthworms in soil tend to bring the pH towards neutrality. The increase in pH is probably due to the pH buffering action of organic molecules produced in the gut of earthworms (Duiker and Stehouwer 2008) in the bioremediation process. It is possible that since the pH is still within the tolerable range (Edwards and Aracon 2006) for the survival and normal functioning of the earthworms, it is not a neutral approach at the end of the study. Sandor and Schrader (2012) observed that as a general trend, the recorded pH values were higher at the end of the experiments compared to the initial start value. Azarpira et al. (2013) reported that the pH value at final stage was increased for all the treatments as compared to initial stage for the vermicomposting process with *E. eugeniae*.

There was a significant decrease (*P* < 0.05) in total organic carbon, sulfate, nitrate, phosphate, sodium, potassium, calcium and magnesium with a decrease towards restoring the normal conditions of the soil to the background levels prior to contamination of soil. Ceccanti et al. (2006) on the combination of earthworms and compost for the ecological remediation of hydrocarbon polluted soil in Finland reported that the chemical parameters showed a decrease in the concentration of total carbon, total phosphorus, and the available carbon and nitrogen in all the treatments. This suggests a progressive degradation of organic compounds, probably including the pollutants (Ceccanti et al. 2006). Although at the end of our study, only phosphate was restored back to the initial background levels of soil.

TOC slightly increased after crude oil contamination of soil and decreased (1.73–0.75) after inoculation with *H. africanus* below the background levels at the end of the experiment. The reason for the further decrease is not clear, but it is probable that the earthworms needed some degree of TOC in the biodegradation process of breaking down pollutant in the crude oil-contaminated soil. Edwards and Arancon (2006) stated that breakdown of compost materials results in low C:N ratios. Sandor and Schrader (2012) reported a significant decrease in soil organic carbon at the end of their experiment. Azarpira et al. (2013) reported that as compared to initial stage, organic carbon decreased in the final stage. The combined action of earthworms and gut microorganisms may be responsible for the loss of organic carbon in the form of carbon dioxide (Prakash and Karmegam 2010). Azizi et al. (2013) also recorded reduction in organic carbon during vermicomposting of sludge. They explained that loss in organic carbon was due to the use of organic carbon by earthworms and microorganisms as source of energy. Similar trend was also reported by Kennette et al. (2002) with *Lumbricusterrestris*, Ceccanti et al. (2006) with *E. fetida* and Ameh et al. (2011) with *L.terrestris*, while Singer et al. (2001), Schaefer et al. (2005), Iordache and Borza (2012) and Ameh et al. (2013) reported higher values.

Sulfate, nitrate, phosphate, sodium, potassium, calcium and magnesium in soil increase significantly after crude oil contamination and decrease significantly after inoculation of earthworms. These soil nutrients are required in the right proportions for the proper functioning of soil fertility for agricultural purposes. At the end of the experiment, these parameters were higher than the initial background levels prior to the experiment. The increase above the background levels is an indication that the earthworm was able to stabilize the soil from a moderately fertile soil to a fertile soil needed for soil flora and fuana. Sinha et al. (2009) reported that the earthworm increases these minerals in the soil by enhancing the soil quality and nutrients. Similar trend was reported by Zavala-Cruz et al. (2013) on crude oil-contaminated soil with earthworms in Mexico. Manyuchi et al. (2013) reported an increase in soil potassium content after 25 days period, but in contrast Sandor and Schrader (2012) reported a significantly lower amount of nitrates compared to those in the corresponding control treatments at the end of their experiment while Iordache and Borza (2011) stated that the concentrations of nitrates and nitrites decreased with 6.93 and 21.43 %, respectively, which are attributed to earthworms, because they consume large amounts of nitrogen in their digestion.

TPH was not detected in the soil prior to contamination. At the end of the study, the TPH in the contaminated soil with earthworms decreased significantly by 68.29 % but for the control without earthworm it only decreased by 11.87 %. There is yet no clear understanding of the biochemical pathway for the bioaccumulation or breakdown of organic compounds in earthworms, but it may not be unconnected with the interactions of chemicals such as enzymes secreted by the earthworms and the decomposer microbial flora associated with the gut of earthworms (Sinha et al. 2010). Schaefer et al. (2005) reported that the TPH concentration decreased significantly in samples with *L. terrestris*, *E. fetida* within 28 days. Schaefer and Filser (2007) also conducted an experiment on the influence of earthworms (*E. fetida, Allolobophora chlorotica*, and *L. terrestris*) and organic additives on the biodegradation of oil-contaminated soil (9500 mg TPH/kg soil dry wt.) in Germany. GC analyses showed that the concentration of TPH was significantly reduced in soils with earthworms compared to the treatments without worms. The efficiency of oil degradation depended on earthworm species. They suggested that earthworms could be applied in the later stages of the bioremediation of even highly contaminated sites, when TPH concentrations and potential toxicity have been decreased to a tolerable or moderate level. Earthworms might be especially useful in in situ remediation by the so-called natural attenuation where the soil is not disturbed by heavy machinery (Schaefer and Filser 2007). Other workers that reported decrease in TPH or crude oil include Schaefer (2001), Ceccanti et al. (2006), Tomoko et al. (2005), Getliff et al. (2002), while Callaham et al. (2002) reported no effect.

Benzene, toluene, ethylbenzene and xylene (BTEX) were below detection limits in the uncontaminated soil. After contamination, the values in the BTEX constituents were in the following descending order; Benzene > Toluene > Xylene > Ethylbenzene in the contaminated soil. The BTEX constituent decreases accordingly after inoculation of earthworms after 30 and 60 days. Only benzene was detected in the soil at the end of the study, while toluene, ethylbenzene and xylene were below detection limit. Hutchins et al. (1992) and Junfeng et al. (2008) also reported a significant reduction in BTEX in petrochemical-contaminated soils. Hutchins et al. (1992) reported average benzene breakthrough of 74.3 ± 5.8 %, 75.9 ± 12.1 %, and 63.1 ± 9.6 % in the columns with limited oxygen, limited oxygen plus nitrate, and nitrate alone, respectively. Junfeng et al. (2008) suggested that all the BTEX substrates could be anaerobically biodegraded to non-detectable levels within 70 days when the initial concentrations were below 100 mg/kg in soil. Toluene was degraded faster than any other BTEX compounds, and the high-to-low order of degradation rates were toluene > ethylbenzene > *m*-xylene > *o*-xylene > benzene > *p*-xylene (Junfeng et al. 2008). Contreras-Ramos et al. (2006) reported PAH removal of 51 % for anthracene, 47 % for benzo(*a*)pyrene and 100 % for phenanthrene in soil with the earthworm *E. fetida*. Krishna et al. (2011) also reported 100 % bioremediation of phenols after 96 h with the earthworms *E.fetida*, *E.eugeniae* and *Anantapur* sp. for phenol concentration of 20, 40, 60, 80 and 100 ppm. Tharakan et al (2004) investigated the biotransformation of polychlorinated biphenyls (PCB’s) in sludge and sediment from the Ralston Street Lagoon (RSL) SUPERFUND site in Gary, Indiana, United States with *E. fetida*. Vermicomposting bioreactors (VBs) were established with mass fractions of contaminated sludge ranging from 10 to 75 %. All sludge concentrations demonstrated around an 80 % reduction in total PCB concentration by the time of termination of the experiment. Singer et al. (2001) achieved 55 % removal of the total soil PCB with *Pheretimahawayana* as compared to only 39 % in identically treated soils without earthworms. Earthworm-treated soils achieved upwards of 65 % PCB degradation at subsurface depths, as compared to 44 % in soils without earthworms.

In bioremediation studies with macrofauna, it is essential to point out the transport and fate of the contaminant. This will give a clear understanding of the bioremediation pathway and the need to take extra steps towards effective removal of the contaminant from the food chain. Although a significant amount of TPH and BTEX were lost from the soil and biodegraded by the earthworm, at the end of the experiment about 57.35 % of TPH and 29.61 % of BTEX were bioaccumulated inside the tissues of the earthworms while 15.01 % TPH and 8.35 % benzene were still remaining in the soil. Tharakan et al. (2004) reported that the bulk of the removal of PCBs from the sludge appeared to be transported into the earthworm biomass. Approximately, 20 % of the PCBs remained in the sludge-earthworm bedding mixture at the time of termination of each of the experimental studies. As suggested by Tharakan et al. (2004), it is entirely possible that a certain percentage of contaminants are hard to access and hence recalcitrant to any transformative activity. In soil where contamination has taken place for years, the aging phenomenon suggested by several workers can be responsible for the hindering or recalcitrant effects of the contaminant, but in laboratory-contaminated soil or lands where pollution occurs within few months, this aging phenomenon is very unlikely. Another possibility is that the earthworms did not have sufficient time and sufficient additional feeding, hence it may be that with sufficient additional resources and longer experiments, more complete biotransformation may occur.

The ability for this species of earthworms to biodegrade contaminant is not known but it is clear from our study that biodegradation and bioaccumulation are processes that are simultaneously taking place in the earthworms. It is also possible that earthworms can tolerate some concentration of contaminant without affecting its regular biological function and it only breakdown contaminant when it is exceeding its tolerance threshold. There is need for further research to shed light on this. In earthworms-contaminated soil, grasses started growing in the media after 20 days while in the control grasses appeared after 41 days.

## Conclusion

Developing an effective strategy for the removal of petrochemicals from contaminated environment is one of the major challenges facing developing countries including Nigeria in her quest for economic development. This research has shown that the earthworm *H. africanus* has the potentials to bioaccumulate and biodegrade hydrocarbons from crude oil-polluted soil. The addition of additives or nutrients for the earthworms will increase and enhance the bioremediation process. These findings suggest that the earthworm could be applied in the later stages of bioremediation, even in highly contaminated soil with crude oil when the toxicity of the contaminant may have decreased or pre-treated to a tolerable level. Earthworm bioremediation does not only remove the contaminant but also enrich the soil by the addition of vermicast after reworking of the contaminated soil in the gut of the earthworm.
